# Human CD4 T-Cells With a Naive Phenotype Produce Multiple Cytokines During *Mycobacterium Tuberculosis* Infection and Correlate With Active Disease

**DOI:** 10.3389/fimmu.2018.01119

**Published:** 2018-05-23

**Authors:** Valentina Orlando, Marco P. La Manna, Delia Goletti, Fabrizio Palmieri, Elena Lo Presti, Simone A. Joosten, Carmela La Mendola, Simona Buccheri, Tom H. M. Ottenhoff, Francesco Dieli, Nadia Caccamo

**Affiliations:** ^1^Central Laboratory of Advanced Diagnosis and Biomedical Research (CLADIBIOR), University of Palermo, Palermo, Italy; ^2^Department of Biopathology, University of Palermo, Palermo, Italy; ^3^Translational Research Unit, National Institute for Infectious Diseases L. Spallanzani, Rome, Italy; ^4^Department of Infectious Diseases, Leiden University Medical Center, Leiden, Netherlands; ^5^Istituto Mediterraneo per i Trapianti e Terapie ad Alta Specializzazione, Palermo, Italy

**Keywords:** CD4 T-cells, naive cells, *Mycobacterium tuberculosis*, tuberculosis, cytokines

## Abstract

T-cell-mediated immune responses play a fundamental role in controlling *Mycobacterium tuberculosis* (*M. tuberculosis*) infection, and traditionally, this response is thought to be mediated by Th1-type CD4^+^ T-cells secreting IFN-γ. While studying the function and specificity of *M. tuberculosis*-reactive CD4^+^ T-cells in more detail at the single cell level; however, we found a human CD4^+^ T-cell population with a naive phenotype that interestingly was capable of producing multiple cytokines (T_CNP_ cells). CD4^+^ T_CNP_ cells phenotyped as CD95^lo^ CD28^int^ CD49d^hi^ CXCR3^hi^ and showed a broad distribution of T cell receptor Vβ segments. They rapidly secreted multiple cytokines in response to different *M. tuberculosis* antigens, their frequency was increased during active disease, but was comparable to latent tuberculosis infection in treated TB patients. These results identify a novel human CD4^+^ T-cell subset involved in the human immune response to mycobacteria, which is present in active TB patients’ blood. These results significantly expand our understanding of the immune response in infectious diseases.

## Introduction

Several studies in experimental mouse models and in HIV-infected patients have underscored the essential role of CD4^+^ T-cells and Th1 immunity in protection against tuberculosis (TB) (1). Moreover, CD4^+^ T-cells producing one, two, or three cytokines (polyfunctional T cells) have been linked to bacterial load and disease activity in humans, as well as to protective TB to vaccine-induced responses in animal models of TB (2–4). Protective immune responses against mycobacterial infection are thought to be dependent on quality, as measured by the capacity of T cells to exert multiple functions, rather than on their magnitude, which is predominantly related to the frequency of antigen-specific T cells with a particular effector function (5, 6).

Detection of antigen-specific cytokine production in individual T cells by intracellular cytokine staining and flow cytometry has been widely used to identify specific subsets of T cells producing different types of cytokines and to correlate their cytokine production patterns with functional phenotypes according to the expression of surface markers.

CD4^+^ T-cells can be divided into at least three different populations: naive (T_N_) referred to as CD45RA^+^CCR7^+^, central memory (T_CM_) as CD4R^−^CCR7^+^ and effector memory (T_EM_) as CD45RA^−^CCR7^−^. T_EM_ cells rapidly respond to re-encounter of antigen, such as in re-infection, whereas T_CM_ cells are long-lived T cells with self-renewal and a homeostatic proliferative capacity that might contribute to their maintenance (7). In several infection models, including TB, T_EM_ cells are expanded during active bacillary replication, while T_CM_ cells are mostly detectable after control and eradication of infection (4, 8), indicating that distinct memory phenotypes and functions are associated with different stages of infection.

In the present study, we performed a broad phenotypic and functional characterization of *M. tuberculosis*-specific CD4^+^ T-cells to evaluate whether distinct T cell functional signatures could be correlated with cell surface memory and effector phenotypes in the different phases of infection and disease.

We used additional surface markers, such as CD28 and CD95, which allow the identification of six CD4^+^ T cell subsets in the peripheral blood of healthy human beings (9). Moreover, we analyzed the expression of other molecules mediating lymphocyte functions, including the CD49d integrin and the CXCR3 chemokine receptor, which is expressed on IFN-γ-producing Th1 cells and allows cells to traffic rapidly to inflamed tissues. Unexpectedly, this approach revealed the existence of a novel human CD4^+^ T cell subset in TB, which is antigen specific, associated with disease activity, and expresses an undescribed phenotype, which explains why these cells have not been detected.

## Materials and Methods

### Human Subjects

Peripheral blood was obtained from 18 adults with pulmonary TB disease (Table 1) recruited from the National Institute for Infectious diseases L. Spallanzani (INMI) in Rome. The majority of TB patients had been vaccinated with bacillus Calmette-Guérin, none had evidence of HIV infection, or was being treated with steroid or other immunosuppressive. Active TB microbiologically diagnosed was defined based on the *M. tuberculosis* isolation from sputum culture. All patients were treated in accordance with Italian guidelines and received therapy for 6 months. Treatment was successful in all participants, as evidenced by the absence of any clinical or radiographic evidence of recurrent disease and sterile mycobacterial cultures at the end of treatment. Peripheral blood from patients was collected within 8 days of specific treatment initiation and, in some patients, after completion of chemotherapy (TB-6). Latent tuberculosis infection (LTBI) individuals reported household or equivalent close contact (work) with smear-positive pulmonary TB patients in the previous 3 months, and scored positive in QuantiFERON Gold In-Tube (QFT IT, Qiagen) with no active TB lesions at chest radiography and had not received any prior preventive therapy (10, 11). Table 1 reports the characteristics of the enrolled subjects included in this study. The study was approved by the Ethical Committee of the University Hospital in Palermo (approval number 13/2013) and of INMI, approval number 72/2015. Informed written consent was provided by all participants.

**Table 1:** Characteristics of enrolled patients.

	Active TB	LTBI	Past TB	Total
*N* (%)	18 (51.4)	7 (20.0)	10 (28.6)	35 (100.0)
Age median (IQR)	39 (29.0–43.5)	37 (28.0–63.0)	44.5 (34.0–51.0)	41 (29.0–45.0)

**Sex**				
Female *N* (%)	5 (27.8)	3 (42.9)	6 (60.0)	14 (40.0)

**Origin**				
West Europe (%)	2 (11.1)	4 (57.1)	2 (20.0)	8 (22.9)
East Europe (%)	10 (55.6)	1 (14.3)	5 (50.0)	16 (45.7)
Asia (%)	2 (11.1)	–	–	2 (5.7)
Africa (%)	3 (16.7)	1 (14.3)	1 (10.0)	5 (14.3)
South America	1 (5.6)	1 (14.3)	2 (20.0)	4 (11.4)

**BCG**				
Vaccinated (%)	16 (88.9)	3 (42.9)	8 (80.9)	27 (77.1)

**QFT-IT**				
Positive (%)	12 (66.7)	7 (100.0)	–	19 (54.3)
Negative (%)	2 (11.1)	–	–	2 (5.7)
Not done (%)	4 (22.2)	–	10 (100.0)	14 (40.0)
Microbiological TB (%)	18 (100)	–	–	18 (100)

*TB, tuberculosis; LTBI, latent tuberculosis infection; BCG, bacillus Calmette-Guérin; IQR, interquartile range; GTF, QuantiFERON-TB GOLD In-tube*.

### Short-Term *In Vitro* Stimulation and Intracellular FACS Analysis

Peripheral blood was collected in heparin tubes, and peripheral blood mononuclear cells (PBMC) were isolated by Ficoll–Hypaque density gradient centrifugation (Euroclone). Recovered PBMCs were counted using a hemocytometer and viability, as tested by trypan blue exclusion, was always >95%. Peptide pools covering the whole sequences of ESAT-6 and CFP-10 *M. tuberculosis* proteins were used, as well as recombinant Ag85B and *Acr* proteins expressed in *Escherichia coli* and purified as previously described (12). PBMCs (10^6^/mL) were stimulated with *M. tuberculosis* antigens at a final concentration of 10 µg/mL or Ionomycin (Sigma, St. Louis, MO, USA, 1 µg/mL final concentration) and phorbolmyristate acetate (PMA, Sigma, 150 ng/mL final concentration). Cells were cultured in a humidified incubator at 37°C with 5% CO_2_ for 6 h in the presence of 5 µg/mL Brefeldin A (Sigma, St. Louis, MO, USA). Following incubation, PBMCs were harvested, washed in PBS containing 1% FCS and 0.1% sodium azide, and then stained with the antibodies (Table S1 in Supplementary Material) in incubation buffer (PBS-1% FCS-0.1% Na azide) for 30 min at 4°C, as indicated by Cossarizza et al. (13).

Subsequently, PBMCs were washed, fixed, and permeabilized (Cytofix/Cytoperm Kit, BD Pharmingen) according to the manufacturer’s instructions and stained for intracellular cytokines with conjugated anti-IFN-γ, anti-IL-2 and TNF-α mAbs (see Table S1 in Supplementary Material). Isotype-matched control mAbs were used. All mAbs were from BD Pharmingen (San Josè, CA, USA). Cells were washed, fixed in 1% paraformaldehyde, and at least 1 × 10^6^ lymphocytes were acquired using a FACSARIA I (BD Biosciences), following gating according to forward and side scatter plots. FACS plots were analyzed using FlowJo software (version 6.1.1; Tree Star, Ashland, OR, USA).

The median background staining level observed in the medium control was 0.02% based on viable CD4^+^ lymphocytes for IFN-γ. A detected antigen-specific response was defined as a response where the percentage of cytokine-positive CD4^+^ lymphocytes was at least twofold above the background observed in the medium control and at the difference between specific response and background level being >0.01%.

The experiments of *in vitro* stimulation were performed on a median PBMC cell count of 1.5 × 10^6^ cells with a minimum of 9 × 10^5^ viable cells upon Ficoll–Hypaque recovery and a maximum of 1.7 × 10^6^ cells being acquired. A minimum of 5 × 10^5^ CD4^+^ T cells were acquired on the viable lymphocyte gate and population of IFN-γ^+^CD4^+^ T cells more than 50 cells were considered for further analysis.

### FACS Analysis of the TCR-Vβ Repertoire

The IOTest Beta Mark TCR-Vβ Repertoire kit (Beckman Coulter, Miami, FL, USA) was used for the assessment of the following TCR-Vβ regions: Vβ1, Vβ2, Vβ3, Vβ4, Vβ5.1, Vβ5.2, Vβ5.3, Vβ7.1, Vβ7.2, Vβ8, Vβ9, Vβ11, Vβ12, Vβ13.1, Vβ13.2, Vβ13.6, Vβ14, Vβ16, Vβ17, Vβ18, Vβ20, Vβ21.3, Vβ22, and Vβ23. This kit includes 8 cocktails, each containing antibodies against 3 different TCR-Vβ regions covering a total of 24 TCR-Vβ regions and approximately 70% of the normal human TCR-Vβ repertoire. In performing the TCR-Vβ repertoire assay, anti-IFN-γ APC, anti-CD3 BV510, anti-CD4 PercPCy5.5, anti-CD45RA BV450, and anti-CCR7 PECy7 were added to the 8 Vβ cocktails. TCR-Vβ antigen usage was analyzed by using general gating strategies based on amine-reactive LIVE/DEAD fixable NIR dead cells exclusion. Selected T-cell populations per each gating strategy were analyzed for TCR-Vβ expression and quantitation. The values were compared to published normal ranges provided by the manufacturer.

### Spanning-Tree Progression Analysis of Density-Normalized Events (SPADE) Analysis

FACS plots (FCS files) were first analyzed by FlowJo and upon gating performed as above described, a new FCS file was generated (exported.FCS3.fcs), which was imported into the SPADE software. SPADE clustering algorithm on the https://Cytobank.org platform was performed in four steps as described by Qiu et al. (14): (a) density-dependent downsampling to equalize the density in the point cloud of cells, (b) agglomerative clustering to partition the point cloud of cells into cell clusters, (c) minimum spanning tree construction to link the cell clusters, and (d) up sampling to map all the cells onto the resulting tree structure. A detailed description of each step is found in Ref. (14). SPADE analysis was performed to visualize single cells, among live CD3^+^CD4^+^ lymphocytes from all patients with active TB. The nodes of the tree reproduce clusters of cells that are similar in terms of marker expression, the color indicates intensity of the different marker staining, presented as arbitrary relative median fluorescence intensity and indicated with a heat map. This tree is constituted by an interconnected cluster of nodes, or clusters of connected nodes, which correspond with phenotypically defined CD4 T cell populations. SPADE uses the size and color of each node to signify the number of cells and median marker expression, respectively.

### Statistical Analysis

Statistical significance (*p* values) of the magnitude of responses was calculated with unpaired two-tailed Student’s *t*-test. Kruskal–Wallis and Mann–Whitney test were used as non parametric test. Data were analyzed using statistical software SYSTAT 11 (Systat Software) or Graph Pad Prism (4.02) (Graph Pad Software).

## Results

### IFN-γ Production by *M. tuberculosis*-Specific CD4 T-Cells With a Naive Phenotype

**Figure 1:**
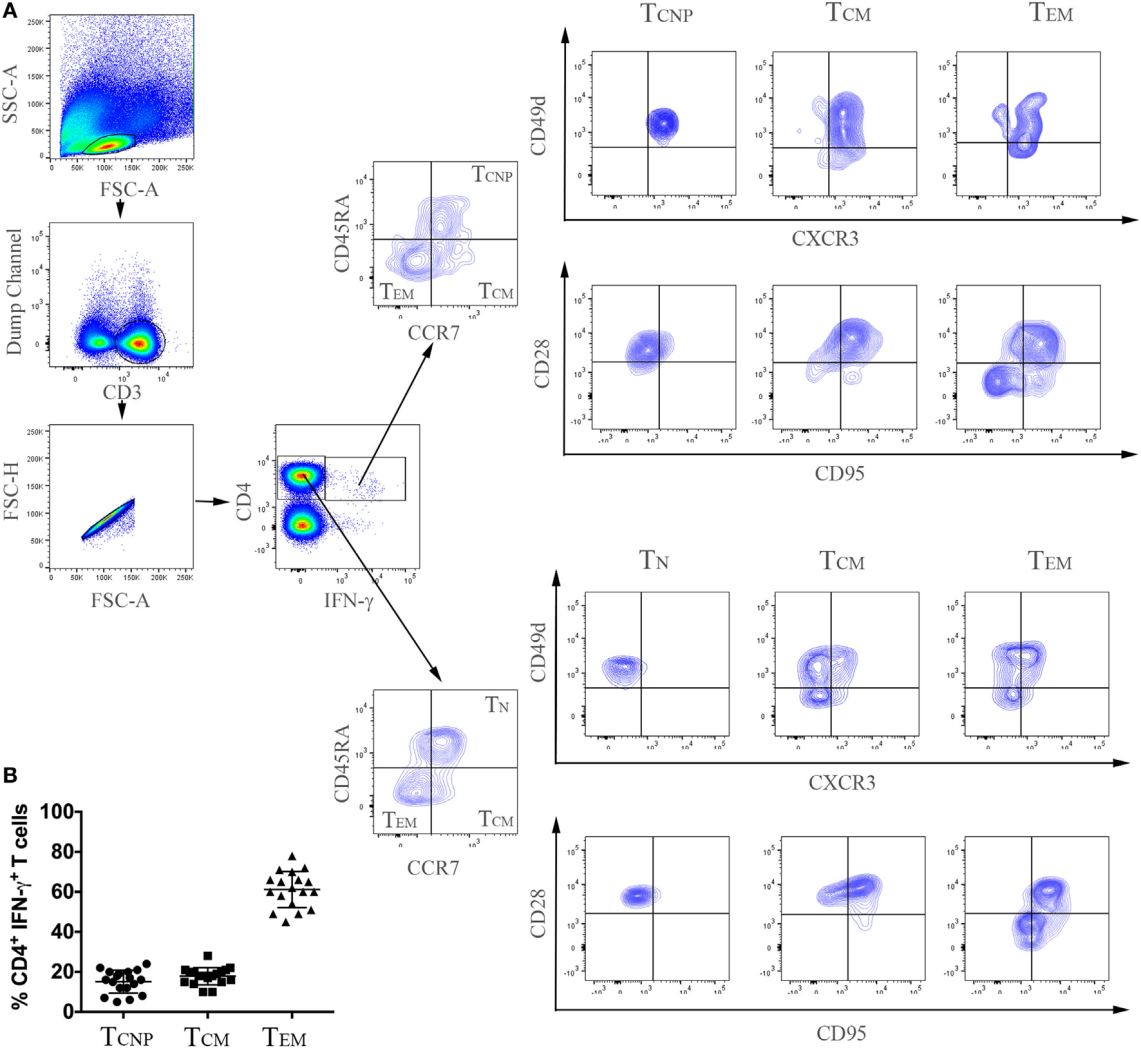
Expansion of CD4^+^ T-cells with a naive phenotype (CD4^+^ T_CNP_ cells) in patients with active TB. **(A)** Flow cytometry analysis of peripheral blood mononuclear cells (PBMC) from one representative TB patient. PBMCs were stimulated with ESAT-6/CFP-10 peptides for 6 h as described under Section “Materials and Methods.” Cells were acquired by a FACSAria I and were gated according to the illustration, as follows: initial gate on dump channel (excluding dead cells, CD19, and CD14 cells) vs CD3^+^, single live cells, and CD4^+^ vs IFN-γ cells. *M. tuberculosis*-specific (CD4^+^IFN-γ^+^) and bulk (CD4^+^IFN-γ^−^) cells were further distinguished in T_CNP_, T_N_, T_CM_, and T_EM_ based on differential expression of CD45RA and CCR7, CD28, CD49d, CD95 and CXCR3 expression was finally evaluated on these differentially-gated populations. **(B)** Cumulative data on the frequencies of ESAT-6/CFP-10-specific CD4^+^IFN-γ^+^ T_CNP_ (CD45RA^+^CCR7^+^CD28^+^, CD49d^+^CXCR3^+^CD95^−^), T_CM_ (CD45RA^−^CCR7^+^) and T_EM_ (CD45RA^−^CCR7^−^) cells in peripheral blood of 18 patients with active TB. Horizontal lines indicate the mean.

**Figure 2:**
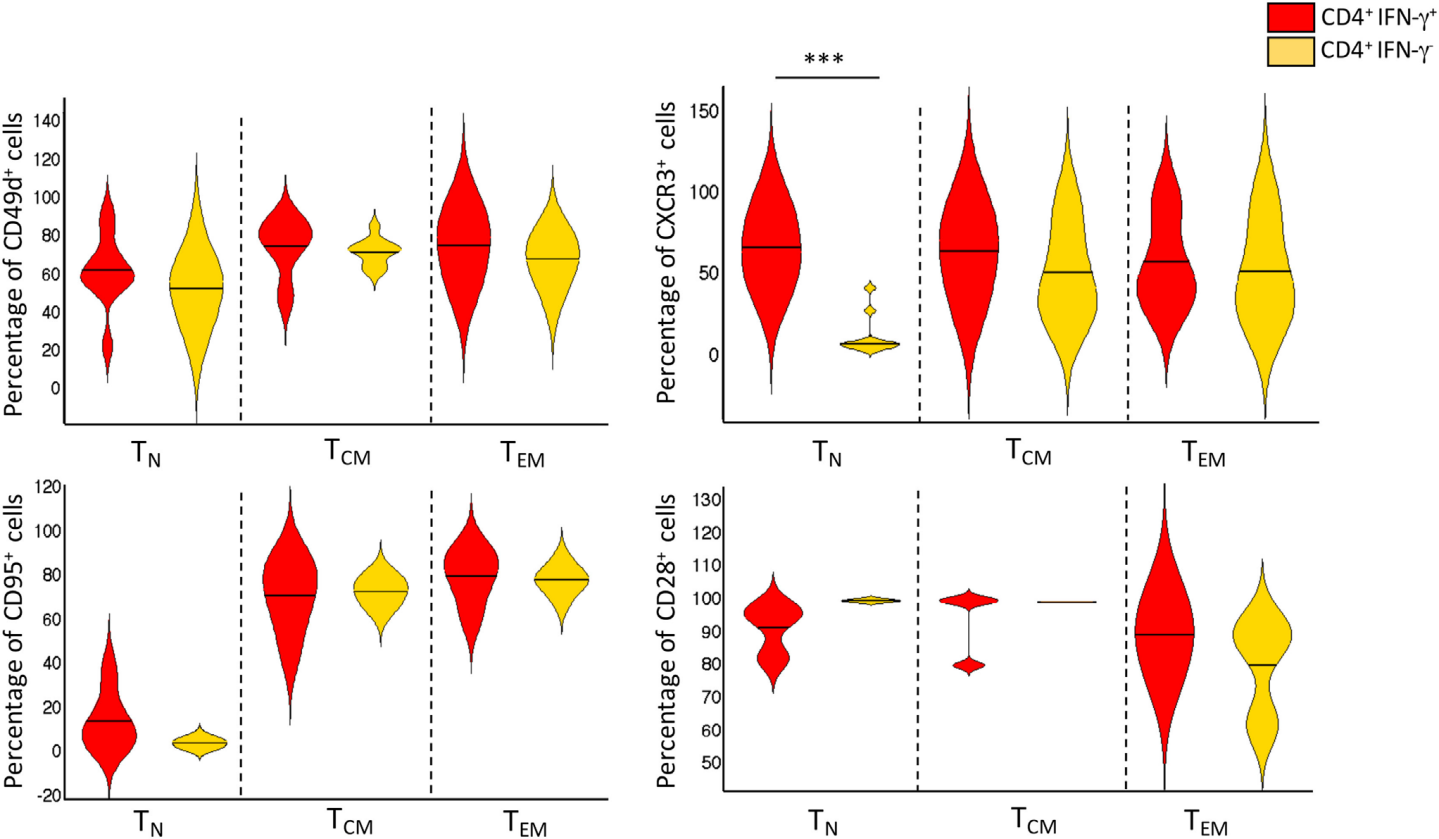
Phenotype distribution and frequencies of CD4^+^IFN-γ^+^ and CD4^+^IFN-γ^−^ T-cell subsets. Violin plot depicting the proportion of cell surface markers expressed by bulk T_N_, T_CM_, and T_EM_ CD4^+^ T cells, and *Mycobacterium tuberculosis*-specific CD4^+^IFN-γ^+^ T_CNP_, T_CM_, and T_EM_ (*n* = 18 active TB patients). Violin plot summarizes the full distribution of the data by density estimation and probability by interquartile range and median (black line). The extremities represent the 95% confidence interval with higher and lower probability respectively. ***p = *p* < 0.001, a value was calculated by Mann–Whitney test.

Peripheral blood mononuclear cells from patients with active TB disease, were stimulated for 6 h with a pool of peptides spanning the whole sequences of *M. tuberculosis* ESAT-6 and CFP-10 antigens and assessed for intracellular interferon-γ (IFN-γ). We then performed flow cytometric analyses to determine memory subset phenotypes of antigen-specific CD4^+^ T-cells, based on their expression of CCR7 and CD45RA (7) (Figures 1A,B). As expected, the majority (~60%) of IFN-γ^+^ CD4^+^ T-cells responding to ESAT-6/CFP-10 in patients with active TB disease lacked surface expression of CD45RA and CCR7, compatible with a T_EM_ phenotype, while 10–28% of IFN-γ^+^ CD4^+^ T-cells expressed CCR7 but not CD45RA, consistent with a T_CM_ phenotype. However, and surprisingly, a sizeable yet variable fraction (5–24%) of CD4^+^ T-cells responding to ESAT6/CFP-10 in patients with active TB disease had a naive (T_N_, CD45RA^+^CCR7^+^) membrane expression phenotype. Figure 1A shows the gating strategy used to select CD4^+^ T cells and comprise of sequential gating, dump channel (excluding dead cells, CD19 and CD14 cells) vs CD3^+^, single live cells, and CD4^+^vs IFN-γ^+^cells, and Figure 1B shows cumulative data of the phenotype distribution of IFN-γ-producing CD4^+^ T cells from 18 patients with active TB disease. To better define this population of CD4^+^ T-cells with an apparent naive phenotype that nevertheless produced IFN-γ in response to *M. tuberculosis* antigens, expression of CD95, CD49d, CXCR3, and CD28 by different subsets of IFN-γ^+^ CD4^+^ T cells was compared to bulk (IFN-γ^−^ cells) T_N_, T_CM_, and T_EM_ cells (Figure 2). Virtually, all *M. tuberculosis*-specific IFN-γ^+^ CD4^+^ T-cells expressed CD28, CD49d, with relatively large proportions also expressing CXCR3, only a very small proportion of CD4^+^T_CNP_ cells expressed CD95. Therefore, and with the exception of CD95, the phenotypic profile of IFN-γ^+^CD4^+^ T_N_-cells was comparable to that of IFN-γ^+^CD4^+^ T_CM_ and T_EM_ cells. Compared to bulk CD4^+^ T_N_ cells, IFNγ^+^CD4^+^ T-cells with the naive surface phenotype could be distinguished by differential expression of CXCR3, with the latter containing a significantly larger proportion of cells expressing CXCR3 (median = 65%), while this was barely detectable in the former (median = 5%).

Thus, this analysis identifies a phenotypically naïve, ESAT-6/CFP-10-specific, IFN-γ^+^CD4^+^ CD45RA^+^CCR7^+^ population that expresses cell surface CD95^+^CD28^+^CD49d^+^CXCR3^+^. These CD4^+^ T-cells with a naive phenotype but producing cytokines were designated T_CNP_ cells (cells with naive phenotype that are able to produce cytokines).

In 5/18 patients with active TB disease, we detected two different populations of IFN-γ-producing CD4^+^ T cells, one IFN-γ^high^ and one IFN-γ^low^: as shown in Figure S1 in Supplementary Material, the IFN-γ^low^ CD4 T-cells coincided with the T_CNP_ population, while the IFN-γ^high^ CD4^+^ T cell population predominantly expressed a T_EM_ phenotype.

To better define the IFN-γ profiles of CD4 T_CNP_ cells, we used the SPADE algorithm, which distinguishes cell subsets by clustering, based on surface antigen expression identified by a color gradient.

**Figure 3:**
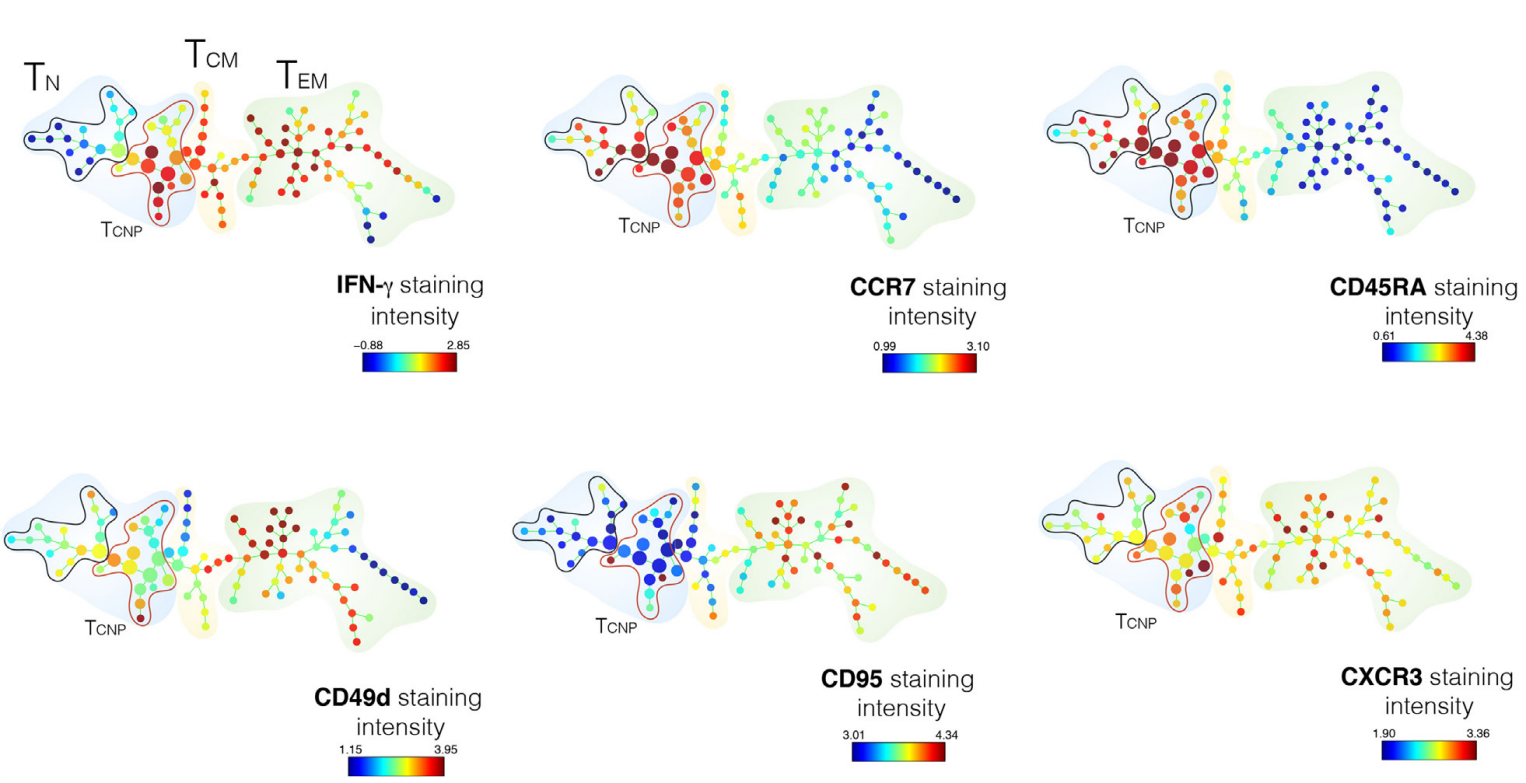
SPADE analysis of CD4^+^T_CNP_ cells. CD3^+^CD4^+^ single cells were used to generate the SPADE tree. CD4 T-cells were analyzed for IFN-γexpression and according to the expression of CCR7 and CD45RA expression. The color patterns indicate that the nodes contained cells that express the same surface marker. The distribution of the major populations is shown for one representative sample. The branching tree is based on the number of cells included in each node and the legend indicates the range of cell per node according to relative median fluorescence intensity of 1 out of 18 TB patients in response to ESAT6/CFP10 after 6 h of *in vitro* stimulation.

As shown in Figure 3, the expression of five T-cell-activation and -differentiation markers (CD45RA, CCR7, CD95, CD49d, CXCR3) distinguished discrete IFN-γ-producing T-cell subsets among PBMCs stimulated with ESAT-6/CFP10 antigens. As expected, the highest IFN-γ staining intensity was detected amongst cell populations, which stained negative for both CD45RA and CCR7 and thus likely corresponding to canonical T_EM_ cells. The T_N_ cell subset, as identified by the expression of CD45RA^+^CCR7^+^, was further split into several nodes, 16 of which corresponded to canonical T_N_ cells and 17 nodes enriched for IFN-γ-producing cells, thus identifying the T_CNP_ subset (Figure 3). The analysis of the mean fluorescence intensity further revealed that CD4^+^IFN-γ^+^ T_CNP_ cells had higher expression of CCR7, CD45RA, and CXCR3, intermediate expression of CD49d and very low, if any CD95 expression. Hence, SPADE analysis confirmed that the phenotypic profile of ESAT-6/CFP10-specific CD4^+^IFN-γ^+^ T_CNP_ cells was markedly distinct from that of T_N_, T_SCM_, T_CM_, or T_EM_ cells (9).

**Figure 4:**
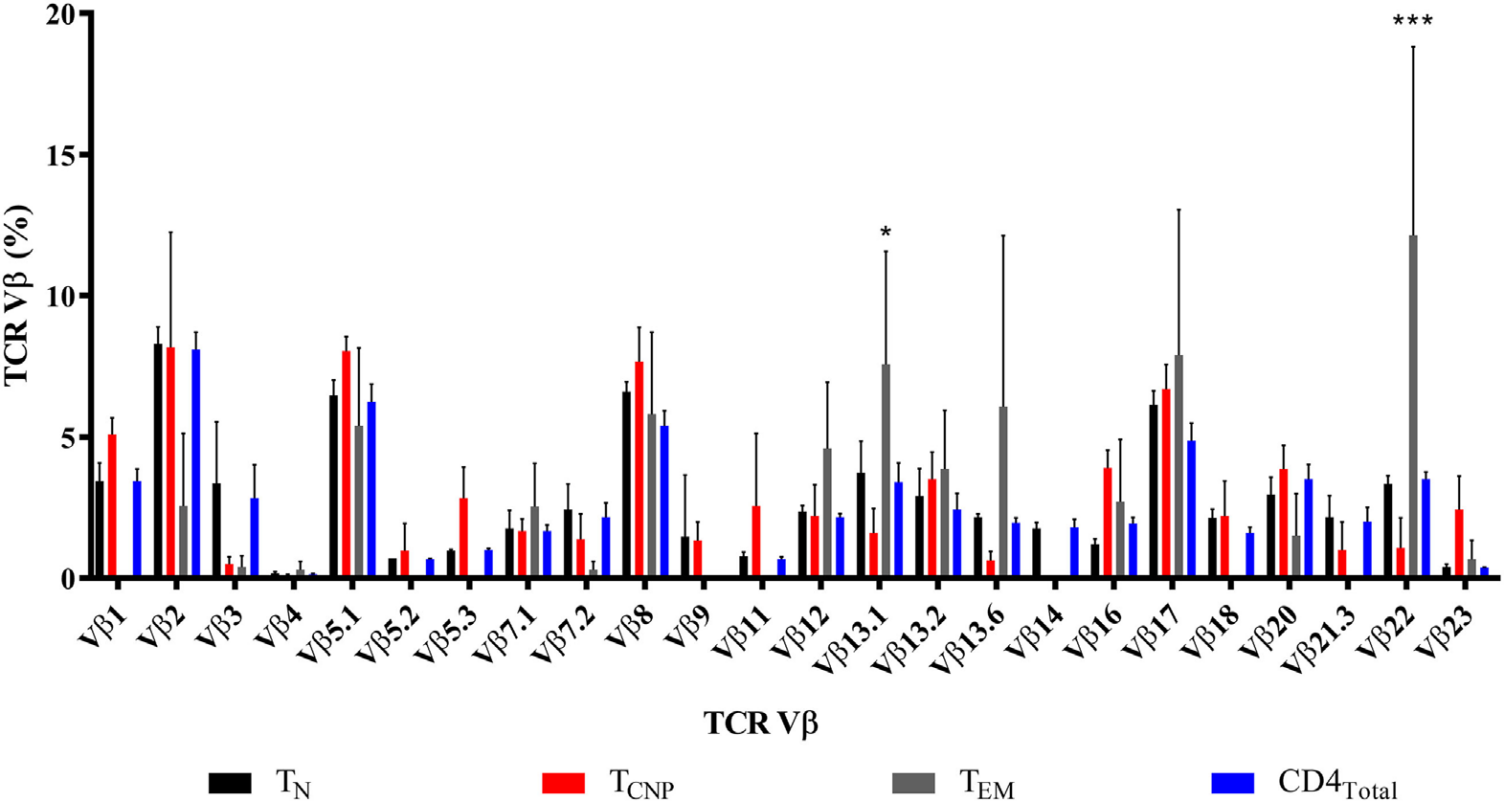
T_CNP_ cells have a broad TCR Vβ repertoire. Distribution of TCR Vβ in each of the 24 TCR Vβ regions assessed (horizontal axis) in the CD4T_N_, CD4T_CNP_, CD4T_EM_, and bulk CD4^+^ T-cells, analyzed on the basis of IFN-γ production and on the expression of CCR7 and CD45RA of three representative patients with active TB. Clonogram represents the mean of the 24 TCR Vβ expression of the three patients. *p < 0.05 when T_EM_ subset was compared to T_CNP_ subset and ***p < 0.001 when T_EM_ subset was compared to the all subsets.

We next assessed the TCR diversity of the CD4^+^ T_CNP_ cells by flow cytometry using antibodies specific for the 24 different TCR β-chain variable (Vβ) regions, which cover 70% of the total Vβ repertoire in three patients. Figure 4 shows that T_CNP_ cells had an overall broad distribution of different Vβ gene segments, but some Vβ families (Vβ5.1, Vβ7.1, Vβ8, Vβ12, Vβ13.2, Vβ16, and Vβ17) were similarly expressed in CD4^+^ T_CNP_ and in the most differentiated CD4^+^ T_EM_ cells, suggesting that the CD4 T_CNP_ cell population might have arisen in response to *M. tuberculosis* antigens that drove the generation of CD4 T_EM_ cells, in a putative linear differentiation model (7). Of note, *M. tuberculosis*-specific IFN-γ^+^CD4^+^ T_EM_ cells had a dominant Vβ22 gene expression and, Vβ13.1, most likely reflecting the emergence of antigen-specific IFN-γ^+^CD4^+^ T effector cells as dominant clones during *M. tuberculosis* infection.

### CD4^+^ T_CNP_ Cells Recognize Multiple *M. tuberculosis* Antigens and Are Polyfunctional

**Figure 5:**
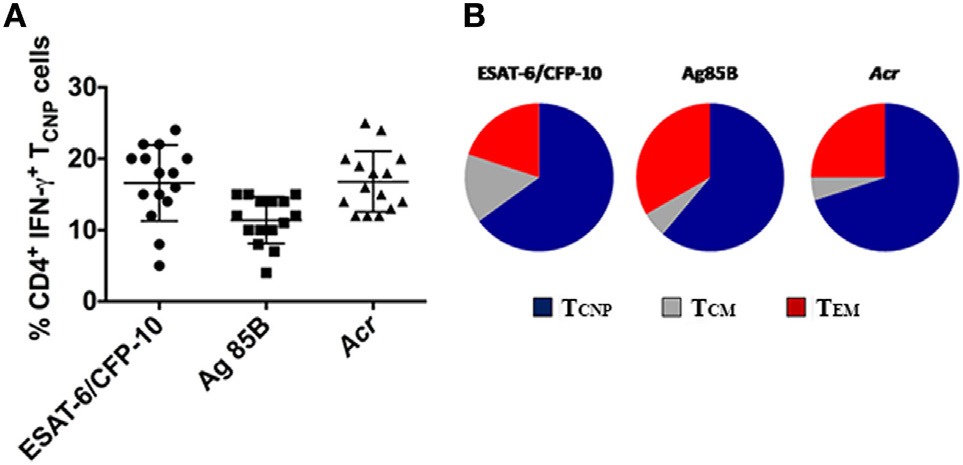
Broad antigen repertoire and polyfunctionality of CD4^+^T_CNP_ cells. **(A)** Cumulative data on the frequencies of ESAT-6/CFP10, Ag85B- and *Acr*-specific CD4^+^IFN-γ^+^T_CNP_ cells in peripheral blood of 15 patients with active TB disease. Horizontal lines indicate the mean. **(B)** peripheral blood mononuclear cells were stimulated with ESAT-6/CFP10 peptides, Ag85B, and *Acr* protein antigens and CD4^+^ T-cell subsets simultaneously expressing IFN-γ, TNF-α, and IL-2 were gated. Triple cytokine-positive CD4^+^ T cells were then gated on the basis of surface marker expression for CD45RA and CCR7 to identify relative proportions of T_CM_, T_CNP_, and T_EM_ subsets.

In addition to ESAT-6/CFP-10, antigen (Ag)85B and α-crystallin (*Acr, HspX, Rv2031c*), which are expressed at different stages of *M. tuberculosis* infection (15) were used to stimulate PBMCs from 18 patients with active TB. As shown in Figure 5, 4–15% of IFN-γ producing CD4^+^ T cells specific for Ag85B in TB patients exhibited the T_CNP_ phenotype, and a similar fraction (12–25%) of IFN-γ-producing CD4^+^ T_CNP_ cells was found in response to *Acr*. These results indicate that CD4^+^ T_CNP_ cells are a common feature of the global immune response to *M. tuberculosis*. In both instances, the proportion of unstimulated CD4^+^ T cells with a T_N_ phenotype that produced IFN-γ (T_CNP_ cells) was <0.01%. Similarly, we consistently failed to detect CD4^+^ T_N_ cells making IFN-γ among PBMC of healthy age- and sex-matched healthy PPD^−^ control individuals (data not shown). However, following 6 h stimulation of PBMCs from healthy donors with ionomycin and PMA, 0.5–4% of CD4^+^ T_N_ cells expressed IFN-γ (data not shown).

The polyfunctionality of CD4^+^ T_CNP_ cells, defined as the simultaneous production of IFN-γ, IL-2, and TNF-α in response to stimulation with ESAT6/CFP-10 peptides or Ag85B or *Acr* proteins, was evaluated further. To this aim, we first gated on CD4^+^ cells expressing IFN-γ, IL-2, and TNF-α and then evaluated their surface memory phenotypes. As shown in Figure 5B, an average 60% of the polyfunctional, triple cytokine positive (IFN-γ, IL-2, and TNF-α) CD4^+^ T cells had a T_CNP_ phenotype, as compared to 15% with a T_CM_ phenotype and 25% with a T_EM_ phenotype. This indicates that the ESAT-6/CFP10-specific CD4^+^ T_CNP_ cells are differentiated polyfunctional T-cells that differ both phenotypically and functionally from the quiescent CD4^+^ T_N_ population.

### CD4^+^ T_CNP_ Cell Frequencies in the Blood Correlate With Active TB Disease

We next determined whether the CD4^+^ T_CNP_ population was stable over time by measuring their frequencies cross-sectionally in samples from 10 patients after completion of anti-TB therapy.

**Figure 6:**
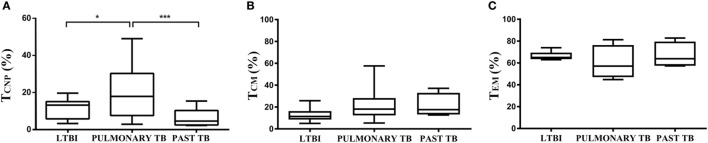
CD4^+^ T_CNP_ cells correlate with active TB disease. Summary cumulative data of the frequency of CD4^+^ IFN-γ^+^ T_CNP_ cells **(A)**, T_CM_ **(B)**, and T_EM_ **(C)** cells in patients with active TB, longitudinally assessed before the initiation of therapy (TB-0), compared to the same patients at the end of therapy, TB-6, and latent tuberculosis infection subjects. The boxes represent interquartile ranges; the line in the middle of the box represents the median, and minimum/maximum values are shown. Data are representative of three independent experiments. *p*-values were calculated by Kruskal–Wallis test. *p < 0.05 and ***p < 0.001 were considered statistically significant.

As shown in Figure 6A, the proportion of CD4^+^ T_CNP_ cells producing IFN-γ significantly decreased in cured TB patients. Similar results were obtained upon stimulation with Ag85B and *Acr* antigens (data not shown). Moreover, we determined whether the CD4^+^ T_CNP_ population was detectable in subjects with latent *M. tuberculosis* infection (LTBI subjects). As shown in Figure 6A, active TB patients had significantly more T_CNP_ cells when compared to LTBI individuals.

For comparison, frequencies of *M. tuberculosis*-specific CD4^+^ T_CM_ (Figure 6B) and T_EM_ cells (Figure 6C) were very similar in the three tested groups, without significant difference among them.

To support the above data, we evaluated frequencies of IFN-γ-producing naïve (CD45RA^+^CCR7^+^CD28^+^CD95^−^) CD4^+^ T cells in a different cohort of 10 patients with active TB before the initiation of therapy (TB-0), compared with blood samples from the same patients taken 6 months later, i.e., at the end of therapy (TB-6). As shown in Figure S2 in Supplementary Material, the frequency of the presumptive T_N_ producing IFN-γ population declined in all tested patients after therapy, strongly supporting previous data obtained in patients with cured TB disease.

All together, these results indicate that *M. tuberculosis*-specific CD4^+^ T_CNP_ cells are a novel T cell subset that is associated with active TB disease.

## Discussion

CD4^+^ T-cell-mediated protection against a wide range of pathogenic microorganisms is dependent on different types of Th cells endowed with distinct effector functions (16).

Our results identify a novel human memory CD4 T cell subset with an unorthodox, T_N_-like phenotype, which is involved in the human immune response to mycobacteria, and which correlates with the presence of active TB disease during infection by *M. tuberculosis*. Following curative TB treatment, the size of this T cell subset significantly decreased, suggesting that these cells are markers of disease activity. Accordingly, LTBI subjects had a lower proportion of this CD4 T cell population in the peripheral blood.

This novel human T cell subset was discovered during our studies to quantitate the proportion of functional CD4^+^ T cells, using standard 6 h *in vitro* stimulation with three different mycobacterial antigens (CFP10/Esat-6, Ag85, and *Acr* proteins) followed by intracellular FACS analysis to determine memory subset phenotypes of antigen-specific CD4^+^ T-cells, based on their expression of CCR7 and CD45RA (7). As expected, the majority of IFN-γ^+^CD4^+^ T cells responding to ESAT-6/CFP-10 in patients with active TB disease had a T_EM_ phenotype, while 10–28% of IFN-γ^+^CD4^+^ T cells displayed a T_CM_ phenotype. Unexpectedly, we detected a sizeable cell population that phenotypically expressed a naive like phenotype yet produced antigen-induced IFN-γ^+^. We termed this population CD4^+^ T_CNP_ due to their capacity to rapidly secrete multiple cytokines while expressing a naive phenotype.

IFN-γ profiles of CD4 T_CNP_ cells were defined in more detail using additional cell surface markers and the SPADE algorithm-tool, which distinguishes cell subsets by clustering on the base of surface antigen expression identified by a color gradient. These analyses revealed that IFN-γ^+^CD4^+^ T_CNP_ cells were further marked by expression of CCR7, CD45RA, CD28, CD49d and CXCR3, and very low, if any CD95 expression. This indicates that the phenotypic profile of ESAT-6/CFP10-specific CD4^+^IFN-γ^+^ T_CNP_ cells is distinct from that of T_N_, T_SCM_, T_CM_, or T_EM_ cells. Moreover, analysis of TCR-Vβ gene usage showed that T_CNP_ cells were not oligoclonal, but displayed highly heterogeneous Vβ gene segment usage. Vβ5.1, Vβ7.1, Vβ8, Vβ12, Vβ13.2, Vβ16, and Vβ17 families were similarly expressed among CD4^+^ T_CNP_ and CD4^+^ T_EM_ cells, suggesting that the CD4 T_CNP_ cell population might have arisen in response to *M. tuberculosis* antigens that drove the generation of highly differentiated CD4 T_EM_ cells (7). Of note, *M. tuberculosis*-specific IFN-γ^+^ CD4^+^ T_EM_ cells showed expanded Vβ13.1 and Vβ22 regions, likely reflecting the emergence of antigen-specific IFN-γ^+^CD4^+^ T effector cells as dominant clones during *M. tuberculosis* infection. Interestingly, previous studies in macaques have demonstrated that the Ag-specific CD4^+^IFN-γ^+^against *M. tuberculosis* is characterized by the emergence of few dominant clones, which undergo major recall expansion and rapidly traffic to the lungs (17). However, additional data are needed due to the limited number of individuals that was tested for TCR Vβ repertoire.

CD4^+^ T cells with a T_CNP_ phenotype accounted for a large proportion, as an average 60% of triple cytokine-positive CD4^+^ T-cells had a T_CNP_ phenotype cells, compared to only 15% expressing a T_CM_ phenotype and 25% expressing a T_EM_ phenotype. This indicates that the ESAT-6/CFP10-specific CD4^+^ T_CNP_ cells are differentiated polyfunctional T cells that differ both phenotypically and functionally from the quiescent CD4^+^ T_N_ population.

Recent studies have shown that polyfunctional T cells provide immune protection in viral infections such as HIV (18) while they are induced in response to a novel tuberculosis vaccine and their role is under investigation (19). They also correlated with protection in a murine *Leishmania* model (5) and in mouse models of TB vaccine-induced protection (20, 21). However, their role during human *M. tuberculosis* infection is controversial and less clear, and these cells do not seem to have the same protection-associated role as observed in chronic viral infections (22). Different authors have recently shown that polyfunctional T cell responses result from a sequential release of cytokines, with very short periods of time in which cells secrete multiple cytokines simultaneously. These results, along with differences in methodological aspects such as culture time, the use of peptide pools or fusion proteins compared to recombinant proteins used, the criteria to select TB patients, might explain the discordant results, and should be taken into account.

Additional work will obviously be needed to address whether the developmental fate of CD4^+^T_CNP_ cells intersects with that of any other subset, and in particular with CD4^+^ T_SCM_ cells, despite the differential expression of certain surface markers, such as CD95 [as shown here and in Ref. (16)]. Our results reported here are in agreement with data on CD8^+^ T cells reported by Nikolich-Zugich and coworkers (23) showing that West Nile virus infection resulted in the expansion of CD8^+^ T cells producing IFN-γ while expressing a naive phenotype, which were termed T_MNP_ cells. These T_MNP_ cells were specifically present in symptomatic subjects, suggesting that their frequency and numbers correlated with the severity of acute viral infection. Finally, also Scriba et al. (24) have identified *M. tuberculosis*-specific CD4^+^ T cells that were induced by primary *M. tuberculosis* infection and displayed a T_SCM_ phenotype while also being capable of producing IL-2, TNF-α, and IFN-γ and, in agreement with the T_CNP_ population we have described here, which predominantly expresses CXCR3.

Whether or not the CD4^+^ T_CNP_ cells here described represent a defined and stable T cell subset (25–27) or rather a transient population, and how it is related or not to T_SCM_ cells remains to be determined. CD4^+^ T_CNP_ cells express CXCR3 and CD49d, which allow them to rapidly traffic to sites of *M. tuberculosis* infection and engage in the control of bacterial replication by virtue of their ability to secrete the type-1 cytokines, IFN-γ, and TNF-α, which are well known players in antimycobacterial protective immune responses. Moreover, as their number also correlates with the presence of active TB disease, they might also serve as a TB specific biomarker of disease as well as a successful response to chemotherapy even if this finding requires to be validated in detail in follow up studies with dense sampling during TB treatment.

## Ethics Statement

The study was approved by the Ethical Committee of the University Hospital in Palermo (approval number 13/2013) and of “L. Spallanzani” National Institute of Infectious Diseases (INMI), approval number 72/2015.

## Author Contributions

Conceived and designed the experiments and wrote the paper: FD and NC. Performed the experiments: CM, SB, VO, and MM. Analyzed the data: FD, NC, VO, and MM. Enrolled the patients and collected the clinical information: DG and FP. Supervised the laboratory collection of the clinical samples: DG. Supervised the paper: TO, SJ, and DG.

## Conflict of Interest Statement

The authors declare that the research was conducted in the absence of any commercial or financial relationships that could be construed as a potential conflict of interest. The reviewer SW and handling Editor declared their shared affiliation.

## Supplementary Material

Figure S1 CD4^+^ T-cells with a naive phenotype (CD4^+^T_CNP_ cells) are found among IFN-γ^low^ CD4^+^ T cells in patients with active TB. Flow cytometry analysis of peripheral blood mononuclear cells from one representative TB patient. Cells were stimulated with ESAT6/CFP10 peptides for 6 h as described in the Section “Materials and Methods” and analysis of cell surface marker expression was differentially performed on the gated CD4^+^IFN-γ^high^ and CD4^+^IFN-γ^low^ populations, demonstrating that the former expresses a T_EM_ phenotype and the latter a T_CNP_ phenotype.

Figure S2 Interferon-γ (IFN-γ)-producing naïve CD4^+^ T cells are linked to active TB disease. Quantification of IFN-γ-producing naïve (CD45RA^+^CCR7^+^CD28^+^CD95^−^) CD4^+^ T cells in a different cohort of 10 patients with active TB disease, longitudinally assessed before the initiation of therapy (TB-0) compared with blood samples from the same patients taken 6 months later, i.e. at the end of therapy (TB-6).

## Abbreviations

*M. tuberculosis, mycobacterium tuberculosis*; TB, tuberculosis; T_CNP_ cells, cytokine-producing T cells with a naïve phenotype.
