# Supplementary figures and images for: Human CD4 T-Cells With a Naive Phenotype Produce Multiple Cytokines During Mycobacterium Tuberculosis Infection and Correlate With Active Disease
Source: Front Immunol. 2018 May 23;9:1119. doi: 10.3389/fimmu.2018.01119 (PMC5974168; doi:10.3389/fimmu.2018.01119)

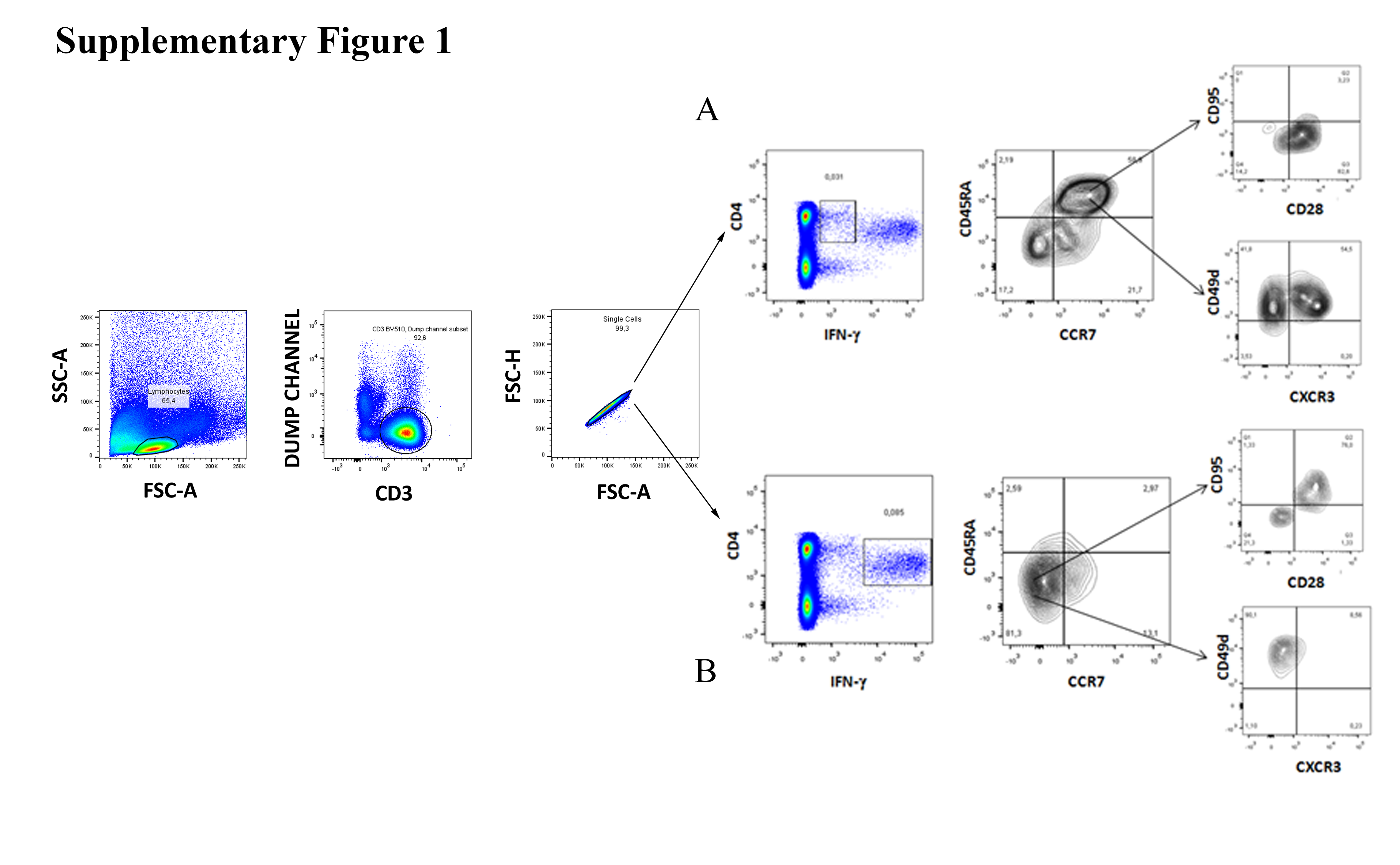

Supplement: Figure S1 — CD4+ T-cells with a naive phenotype (CD4+TCNP cells) are found among IFN-γlow CD4+ T cells in patients with active TB. Flow cytometry analysis of peripheral blood mononuclear cells from one representative TB patient. Cells were stimulated with ESAT6/CFP10 peptides for 6 h as described in the Section “Materials and Methods” and analysis of cell surface marker expression was differentially performed on the gated CD4+IFN-γhigh and CD4+IFN-γlow populations, demonstrating that the former expresses a TEM phenotype and the latter a TCNP phenotype. [file image_1.tif]

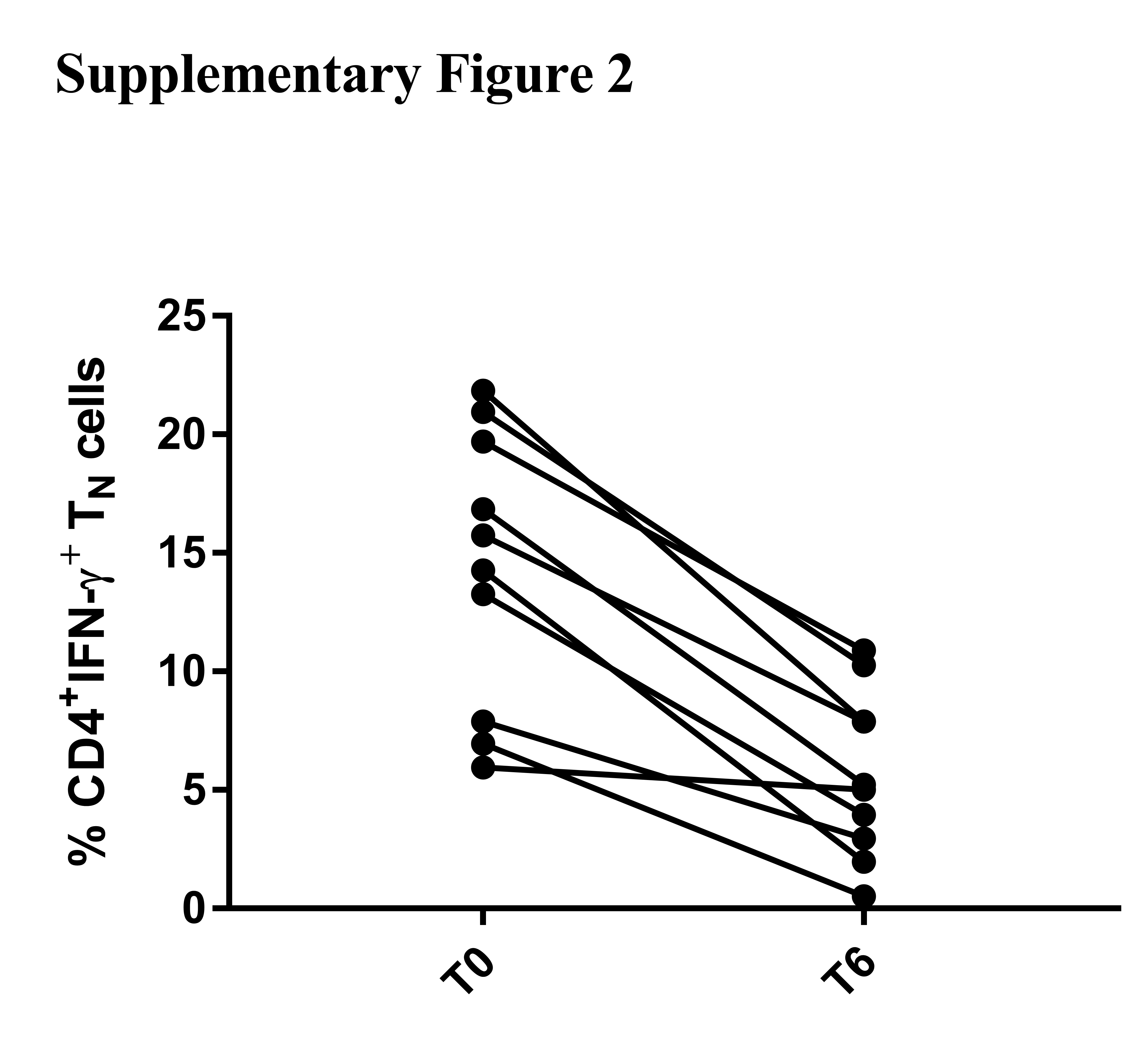

Supplement: Figure S2 — Interferon-γ (IFN-γ)-producing naïve CD4+ T cells are linked to active TB disease. Quantification of IFN-γ-producing naïve (CD45RA+CCR7+CD28+CD95−) CD4+ T cells in a different cohort of 10 patients with active TB disease, longitudinally assessed before the initiation of therapy (TB-0) compared with blood samples from the same patients taken 6 months later, i.e. at the end of therapy (TB-6). [file image_2.tif]
