# Supplementary material for: Human CD4 T-Cells With a Naive Phenotype Produce Multiple Cytokines During Mycobacterium Tuberculosis Infection and Correlate With Active Disease
Source: Front Immunol. 2018 May 23;9:1119. doi: 10.3389/fimmu.2018.01119 (PMC5974168; doi:10.3389/fimmu.2018.01119)
Supplement: Supplementary file 3 [file table_1.docx]

**Supplementary table 1:** **Antibodies used to detect surface markers and cytokines expression profiles of CD4^+^ T cells.**

| **Marker** | **Clone** | **Manufacturer** | **Fluorochrome** | **Sample Type** | **Staining** |
| --- | --- | --- | --- | --- | --- |
| CD3 | HIT3a | BD biosciences | BV510 | Cryopreserved PBMC | Surface |
| CD4 | RPA-T4 | BD biosciences | PerCPCy5.5 |  |  |
| CCR7 | 3D12 | BD biosciences | PE |  |  |
| CD45RA | HI100 | BD biosciences | FITC |  |  |
| CD95 | DX2 | BD biosciences | PE-Cy7 |  |  |
| CD28 | CD28.2 | BD biosciences | BV421 |  |  |
| CD49d | 9F10 | BD biosciences | PE-CF594 |  |  |
| CXCR3 | 1C6 | BD biosciences | BV421 |  |  |
| CD19 | SJ25C1 | BD biosciences | APC-Cy7 |  |  |
| CD14 | MφP9 | BD biosciences | APC- Cy7 |  |  |
| IFN-γ | 4S.B3 | BD biosciences | APC |  | Intracellular |
| IL-2 | 5344.111 | BD biosciences | BV421 |  |  |
| TNF-α | MAb11 | BD biosciences | PE-CF594 |  |  |
| LIVE/DEAD | (Ref 423106) | Biolegend | NIR |  | Surface |
